# Compressive Sensing for Dynamic XRF Scanning

**DOI:** 10.1038/s41598-020-66435-6

**Published:** 2020-06-19

**Authors:** George Kourousias, Fulvio Billè, Roberto Borghes, Antonio Alborini, Simone Sala, Roberto Alberti, Alessandra Gianoncelli

**Affiliations:** 1 Elettra – Sincrotrone Trieste S.C.p.A, 34149 Basovizza, Trieste, Italy; 2 XGLab srl, Bruker Nano Analytics, 20134 Milan, Italy; 3 Present Address: MAX IV Laboratory, Lund University, 22100 Lund, Sweden

**Keywords:** Scientific data, Imaging techniques, Microscopy, Information theory and computation

## Abstract

X-Ray Fluorescence (XRF) scanning is a widespread technique of high importance and impact since it provides chemical composition maps crucial for several scientific investigations. There are continuous requirements for larger, faster and highly resolved acquisitions in order to study complex structures. Among the scientific applications that benefit from it, some of them, such as wide scale brain imaging, are prohibitively difficult due to time constraints. However, typically the overall XRF imaging performance is improving through technological progress on XRF detectors and X-ray sources. This paper suggests an additional approach where XRF scanning is performed in a sparse way by skipping specific points or by varying dynamically acquisition time or other scan settings in a conditional manner. This paves the way for Compressive Sensing in XRF scans where data are acquired in a reduced manner allowing for challenging experiments, currently not feasible with the traditional scanning strategies. A series of different compressive sensing strategies for dynamic scans are presented here. A proof of principle experiment was performed at the TwinMic beamline of Elettra synchrotron. The outcome demonstrates the potential of Compressive Sensing for dynamic scans, suggesting its use in challenging scientific experiments while proposing a technical solution for beamline acquisition software.

## Introduction

X-ray Fluorescence (XRF) spectroscopy and imaging are well established techniques used in a wide range of scientific applications^1–3^. In the case of imaging, the XRF emission signal is acquired for each position of a scanned sample, resulting, after suitable analysis, into a set of elemental maps. This combination of elemental information with spatial distribution provides important information on the analysed areas but being based on a scanning acquisition mode implies that large maps (>1 M points) may require long acquisition times. In the soft X-ray regime also known as Low Energy XRF (LEXRF) the acquisition times are even longer due to the low fluorescence yield below 2 keV. LEXRF on the other hand is crucial for studies and applications that probe light elements, such as C, N, O, Na, Mg, Al, Si and P, important in biological systems, and transition metals (Mn, Fe, Ni, Co, Cu and Zn). Such applications are often biomedical and pose challenges when it comes to imaging large area samples like brain tissues^4–7^ or monitoring fast dynamic phenomena like electrochemical processes^8,9^. Improvements in LEXRF detectors and soft X-ray sources together with advanced instrumentation (i.e. better vacuum conditions, larger detection solid angle, and better motor stages) pave the way for such large scans that may enable new science. Still, additional approaches may be needed as the aforementioned improvements may not translate directly into reduced measurement times. Table 1 shows typical acquisition times and scan range in pixels for the LEXRF system installed at the TwinMic beamline^10^ (case a). It also shows that by increasing the number of pixels by 100 times (from 100 × 100 to 1000 × 1000), even by reducing the acquisition time by 3 times (case b), the total duration of the scan can reach forbiddingly high numbers compared to the duration of a beamtime experiment. However if the acquisition is based on sparse and compressive sensing approaches such those this paper is proposing (case c), the total measurement can become feasible. Thus, this manuscript suggests reduced and selective ways for acquiring LEXRF maps, which can be obviously applied to any energy range. It is based on Compressive Sensing^11,12^ which is an emerging and very effective technique for reconstruction from a relatively small number of data samples without compromising the imaging quality; indeed in our case it allows performing scans of a dynamic nature where it is possible to skip points (sparse) and acquire with variable parameters (i.e. acquisition time). Such an approach may lead to a substantial reduction of the required time. This reduction in terms of time, combined with other technological and scientific advances, like new detectors, can produce maps of very large areas that could not be acquired with the traditional ways. This paper reports the results of the suggested method applied in a series of LEXRF acquisitions at the TwinMic synchrotron spectromicroscopy beamline^10^ of Elettra Sincrotrone Trieste (Trieste, Italy). A flexible and modular beamline control system was used^13^ in order to perform Compressive Sensing scans while post-processing software has been developed for the reconstruction of the sparse maps into traditional ones that can be processed with standard analysis software. The used detector system is based on a 8 SDD setup^14,15^ with a novel multi-channel analyser^16^ (DANTE digital pulse processor, XGLab Bruker).

**Table 1:** The time required at TwinMic for a typical LEXRF scan of 100 × 100 positions and 3 s acquisition per pixel (a) is increasing prohibitively for scans of larger sizes (b). The proposed compressive sensing method can render such scans feasible (c).

	Width x Height (total scan positions)	Acquisition time (dwell per point)	Total scan time (excluding overhead)	Feasibility
a	100 × 100 (typical scan)	3 s	8.3 hours	normal
b	1000 × 1000 (megapixel range)	1 s (with new SDDs)	11.5 days	impractical
c	1000 × 1000 (proposed method)	Variable 0.5–1 s in sparse scanning at 15%	10 to 20 hours	feasible

## Results and Discussion

### Sparse scans and masking

**Figure 1:**
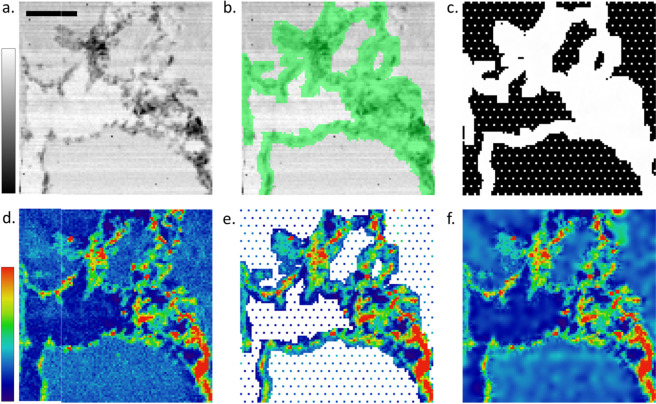
A rapidly acquired STXM map (**a**) (80 µm × 80 µm, 50 × 50 pixels, 20 ms dwell time/pixel, scale bar = 20 µm) is used to create a mask (**c**) which is dense only in the regions of interest (green areas in **b**). A sub-sampled sparse XRF acquisition (**e**) is approximately 3 times faster than a full one (**d**). When the sparse acquisition is reconstructed through biharmonic in-painting (**f**) it can be processed with the usual XRF workflows producing similar results. In this specific case Panels d, e and f show Si XRF signal collected at 1.95 keV on a foraminifera section over an area of 80 µm × 80 µm, with 1.6 µm step size and 3 s acquisition time/pixel.

Typical scanning assumes the acquisition of XRF spectra at positions belonging to a regular cartesian grid. This simplifies various processes but most notably allows for the rectangular representation of the data in the form of images or volumes. The first type of reduced acquisition we implemented is Sparse scanning coupled with masking. It consists of two steps: i) manually selecting certain features of the sample that need to be scanned (Masking) at a certain spatial resolution and ii) acquiring data at sample stage steps larger than the focal spot size of the X-ray beam in the remaining masked areas (Sparse scanning) while at high resolution in the regions of interest. It should be noted that the density of the Sparse scan can also be variable. The workflow of the proposed method is shown in detail in Figure S1 in Supplementary Data through a suitable flowchart scheme. The selection (or masking) is performed through a simple interface (e.g. touch screen, pen stylus, smartphone) on preview data such as a faster scan or scanning transmission X-ray microscopy (STXM) map. In order to assemble the sparse and masked data so that they can be processed, fitted and visualised by software (i.e. PyMca^17^) in a suitable elemental map, we reconstruct it into a dense matrix by using a suitable in-painting technique^18,19^. It should be noted that PyMca^17^ allows for easy visualisation of sparse data without the need to densify the map (Mask Scatter View); still in-painting techniques may improve the visualisation at processing cost. The in-painting fills the missing values of the Sparse scan by suitable interpolation based on the neighbouring XRF data. The result is a 3-dimensional array (width, height, energy) compatible with the standard processing workflows. A visual example of the described method is presented in Fig. 1, where a standard XRF map (Fig. 1d), in this case of Silicon, acquired point by point in a regular square scan on a foraminifera shell is compared with the same scan acquired with the proposed approach (Fig. 1e), based on a mask (Fig. 1b) defined from a previously acquired absorption image (Fig. 1a), and finally reconstructed (in-painted; Fig. 1f) to resemble a full one. The mask is also extended by a set of sparse scan points (Fig. 1c), in order to sample the areas outside the regions of interest. The reported example demonstrates the advantage of the method. The less interesting areas (in this case the ultralene foil, that is the sample support, and the paraffin embedding medium) are under-sampled while the regions of interest (the shell sections) are scanned with a suitable spatial resolution, allowing to provide the same scientific information with a reduced acquisition time. For this particular system the measurements that lead to Fig. 1d required 6 hours, while the ones that produced Fig. 1f just 2 hours.

### Conditional scans and multimodal acquisitions

**Figure 2:**
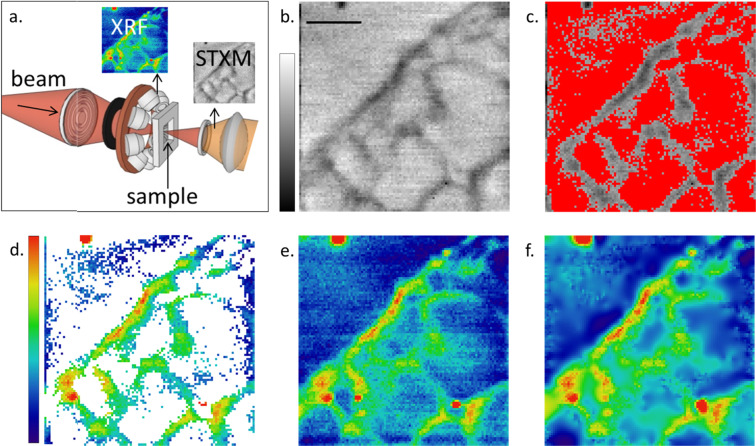
A dual XRF + STXM setup (**a**) allows for using the fast STXM modality (**b**) (80 µm × 80 µm, 50 × 50 pixels, 20 ms dwell time/pixel, scale bar = 20 µm) as a decision factor for performing a slower XRF acquisition (**d**). The scan positions in red (**c**) are above a transmission threshold resulting to a fast 66% sub-sampled XRF acquisition (**d**). The in-painting reconstruction (**f**) of the sparse map (**d**) is comparable to a slow full acquisition (**e**). In this case Panels c, d and f show Si XRF signal collected at 1.95 keV on a soybean root section over an area of 80 µm × 80 µm, with 1.6 µm step size and 3 s acquisition time/pixel.

Some XRF instruments are embedded in more complex systems, using additional detectors which allow them to operate in a multimodal way. The TwinMic beamline endstation for instance allows for the concurrent acquisition of XRF and STXM data^20^ (Fig. 2a). In a Conditional Scanning mode, the system decides on-the-fly whether it should acquire XRF data for a given point according to the transmission signal of that point. Note that in many setups, STXM data may be collected 2 orders of magnitude faster than XRF. Such a conditional scanning represents a case of Compressive Sensing by using a fast probing microscopy technique of less costly nature, in terms of time, to decide whether to acquire data in that specific point. The result is processed in a similar manner to the Masked scans where the missing values are interpolated in order to reconstruct a complete matrix.

Figure S1b in Supplementary Data illustrates in detail the proposed acquisition method by a suitable flowchart while Fig. 2 depict a successful example of it. Based on the simultaneous acquisition of an absorption image (2b) and on the choice of an absorption threshold, which discriminates the sample from the support and the embedding medium (2c), the XRF signal, of Silicon in this case, is acquired only on specific areas (2d), allowing to speed up the acquisition compared to the standard full area (2e). For this specific example, where a soybean root section^21^ was imaged, the proposed method allowed to reduce the measurement time by around 66%. In order to obtain a complete image, an in-painting reconstruction is performed *a posteriori* (Fig. 2f), as described in the previous section.

### Dynamic XRF scanning

Traditional XRF scans generate maps that are processed as a whole. Step size, spot size, and acquisition time are common parameters across the whole map: they are usually set at the beginning of the acquisition and kept constant for the whole measurement. The proposed Dynamic XRF strategy allows for any parameter to be variable in each position of the scan. The variation (e.g. of acquisition time) at a given scan position depends on the actual XRF signal that is collected there in real-time. During the experiment this compressive sensing approach allows to decrease the acquisition time in the areas of the map which have for instance the composition of the background (i.e. ultralene or Si_3_N_4_) and increases it for better statistics for regions containing the elements of interest. The resulting map requires a normalisation of the acquisition time of each pixel prior to fitting it with the traditional software. The workflow of the proposed method is presented in detail in Figure S1c in Supplementary Data through a general description of the procedures involved. The objective is to increase the acquisition time for the scan positions where a predefined element of interest is present.

**Figure 3:**
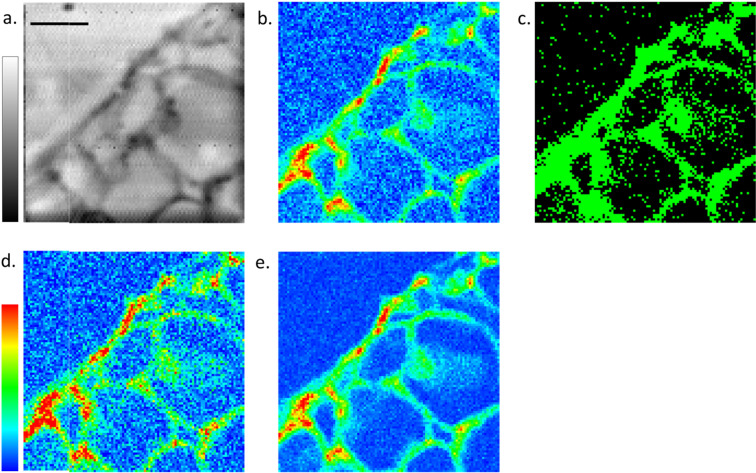
(**a**) STXM absorption image of a 7 µm thick soybean root section where the XRF maps were collected (80 µm × 80 µm, 100 × 100 pixels, 40 ms dwell time/pixel, scale bar = 20 µm). (**b**) Resulting dynamic XRF scan where the point indicated in black in panel (**c**) where acquired with 1 s acquisition time, while the one in green with 7 s, all normalised to 1 s acquisition time. (**c**) According to the pre-set XRF threshold the points in black were acquired at 1 s and the ones in green at 7 s. Full XRF map acquired with 1 s/pixel (**d**) and 7 s/pixel (**e**). In this specific case Panels b, d, and e show Na XRF signal collected at 1.95 keV over an area of 80 µm × 80 µm, with 800 nm step size.

Figure 3 showcases an example of the proposed approach on a section of a soybean root specimen^21^ acquired with submicrometric spatial resolution. Figure 3a depicts the absorption image of the analysed area, to highlight its morphology, even though it is not used at any stage of the proposed method. The dynamic scan acquisition assumes the definition of an XRF signal threshold for a specific element. This can be retrieved by basic signal integration of more sophisticated fitting. For each position in the scanned area, a fast XRF acquisition is used to decide dynamically whether to acquire a longer XRF signal for better statistics on that position. For this specific case a ROI window was selected across Na peak in the XRF spectrum and the threshold was applied to this signal. The fast acquisition was set to 1 s while the longer one to 7 s. Where the threshold was met the acquisition was set to 7 s instead of 1 s. By doing so a set of XRF data were collected at variable exposure times (1 and 7 s) that after a suitable normalisation were reconstructed into a regular map (Fig. 3b). In order to estimate the reduction in time compared to acquiring a full 7 s/pixel map, Fig. 3c shows in black the pixels which were acquired at 1 s and in green the ones at 7 s. Considering that 32% of the areas was acquired at 7 s while the remaining one at 1 s, the dynamic scan required 43% of the total time (full XRF map at 7 s). For comparing and validating the results, full Na maps were acquired at 1 s (Fig. 3d) and 7 s (Fig. 3e). The advantage of the proposed method is evident by comparing Figs. 3b,e, where the Na signal is the same on the cell walls of the root, i.e. the regions of interest, while noisy (same as in Fig. 3d) in the remaining ones. This means that maps with similar information on Na can be obtained by saving 57% of the total time for this given sample.

**Figure 4:**
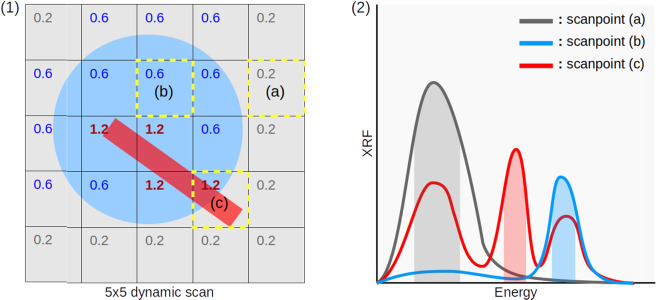
Panel (1): a 5 × 5 pixel XRF scan of a sample containing three characteristic distinct elements (grey, blue, red). The dynamic modality is set to expose longer for the Red element (1.2 s) and less for the Blue (0.6 s) and Grey (0.2 s). Panel (2): XRF spectra of three scanpoints (panel 1: a,b,c in yellow). The presence of Red and/or blue XRF peak in (**c**) allows for varying dynamically on-the-fly a suitable acquisition time.

The proposed Dynamic XRF strategy could also by extended by considering more than one chemical element of interest, as proposed in Fig. 4, where a simplified region scanned in 5 × 5 steps is shown in panel 1. The aim is to increase the acquisition time for the scan positions where objects characterized by predefined chemical elements are present. The grey areas represent the background substrate while the blue circle and the red rectangular region are the features of interest, with the red region being a higher priority one. This approach assumes that the regions of interest in the specimen are distinguished by at least one specific characteristic element which is not present in the background or in less interesting regions. Panel 2 depicts possible spectra of the three areas, grey, red and blue, plotted with the same colors as the corresponding areas. The element indicated with the red ROI (c) is not present in the grey and blue areas therefore it can be used as fingerprint for the red region in panel 1. This simplified example of a dynamic scan assumes that higher XRF statistics are required for the red element regions without knowing *a priori* their location in the scan. With this strategy the acquisition time can be increased for instance to 1.2 seconds when the red “peak” (panel 2) is present while the grey areas of the background are fast scanned at 0.2 s. Since the blue region is still an area of interest but of less importance than the red region, one can decide to increase the acquisition time for instance to 0.6 s when the red peak is absent but the blue one is present. This simple example can be easily extended to more complex methods where ratios of XRF signals are taken into account. Such strategies can be especially useful in XRF scans where there is a hypothesis for the existence of a trace element in an unknown or limited region of the sample.

### Current perspective and future directions

**Figure 5:**
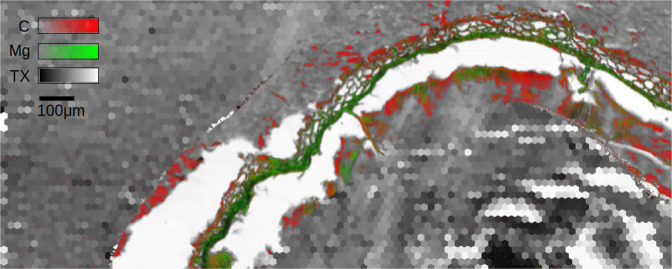
0.4MPixel Sparse scan and Masking combined with a Conditional scan and multimodal acquisition on a cane root section sample. The STXM data (grey) (2000 µm × 800 µm) are acquired with a dense sampling (2 µm step size) along the border of the cane root section while in a sparse way (20 µm step size) in the remaining masked areas. The XRF data have been collected in a similar reduced manner (C in red, Mg in green) and are displayed overlapped with the STXM image. A traditional/complete acquisition (2 µm step size) on the total area would require a measurement time 3 times longer.

The proposed methods [sec. 1,2,3] for Compressive Sensing in XRF scans, other than rendering new experiments feasible (large areas, faster scans, more samples), may pave the way for additional advances both scientific and technological. For instance the authors and their associated labs are outlining a pump-probe inspired experiment where the XRF acquisition is subsequent to an excitation (i.e. sample heating through radiation); since these kinds of experiments have specific timing to track transition or changes in the samples, they could really benefit from the proposed approaches. Fast XRF mapping of large sample areas at high spatial resolution could provide multi-scale insights, since it could be applied to different length-scales (from mm to nm) and promote high impact research in multiple scientific fields from material science to medicine, archaeometry and food science. Elemental maps of large areas (mm) of such samples and nanometric resolution could be acquired faster with the dynamic scanning strategies proposed in this manuscript. Moreover, the proposed methods can be applied not only singularly but also as a combination of different ones and they could benefit from Machine Learning techniques. For instance Machine Learning can be used both for in-painting methods (filling in the missing XRF points in Sparse scans) and for altering the motor steps of the scan (position jumping). Figure 5 reports an example of a successful combination of methods 1 and 2 on a cane root section. A preliminary mask was selected to cover the root section borders adopting an in-painting strategy for the remaining areas. The mask was deliberately left wider than the actual root borders to compensate for possible sample drifting during the scan. In the non-masked areas method 2 was applied in order to acquire the XRF signal only for specific absorption signals, in this case the point corresponding to the root. The deployment of method 2 in the non-masked region assured a faster and safer (free from possible sample drifting) acquisition, then deploying method 1 alone. Figure 5 depicts the overlap of STXM and LEXRF scans over an area of a 2 mm × 0.8 mm of the cane root section. The scan resolution using the techniques of this paper varied from 20 to 2 microns on the masked and the finest resolved areas, respectively. This allowed for examination of C and Mg on the border of the root by performing a scan 3 times faster than that of a traditional dense one.

Eventually the proposed techniques can be used for fly scans (the currently used DANTE electronics permits it^16^) and may be extended to variable velocity scans. The increase in speed could be also useful for XRF tomography experiments that tend to be very challenging and time-consuming. An extension of conditional scans and multimodal acquisitions [sec. 2] is the use of phase contrast information^22,23^ rather than simple STXM transmission as a decisive factor for the XRF acquisition. Finally XRF topography artifacts are a topic of research^24–27^ and is only natural to use the phenomenon in the context of Dynamic XRF scanning [sec. 3].

## Conclusions

This paper suggests a series of methods for performing XRF scans in dynamic ways. It is a Compressive Sensing approach where the data are collected in a reduced manner and later are reconstructed to standard representations. This paves the way for larger and faster scans enabling new science. Through a series of beamtime experiments and suitable software and instrumentation modifications, the feasibility of such an approach was demonstrated. The proposed techniques are presented in three main categories; Sparse scans & Masking, Conditional scans & multimodal acquisitions, and Dynamic XRF scanning. Examples of reduced raw data and final reconstructions are shown and FAIR data and codes are set accessible to the community through a public repository [10.5281/zenodo.3688316]. Finally certain future perspectives have been presented and hint on the use of Machine Learning for the decision-making and reconstruction of missing data component of the methods. The proposed techniques can be combined with other advances on instrumentation and detectors but it should be noted that they introduce an additional challenge on the post-processing reconstruction phase of the reduced data. The expected impact of this research is the use of powerful synchrotron LEXRF for the study of a high number of biological samples of large areas at very high resolutions. Naturally the potential applications can be across different fields from biology to material science in different energy ranges and potentially could include other scanning microscopy techniques (SEM, FTIR, Ptychography etc).

## Materials and Methods

### Experimental

The LEXRF experiments were performed at the TwinMic beamline^12^. The end-station was operated in Scanning Transmission Mode (STXM) where the monochromatic beam is focused on the sample plane by means of zone plate diffractive optics while the specimen is raster-scanned across the microprobe^18^. For the present experiments a gold 600 μm diameter zone plate with 50 nm outermost zone was used producing a microprobe with 1.6 μm or 800nm diameter size at 2 keV. The samples were raster-scanned with a step size of 20 or 2 micron.

The specimens shown in Figs. 1 and 2 are 10 micron thick sections of foraminifera shells deposited on ultralene foils. The sample depicted in Fig. 5 is a 10 micron thick cross section of a cane root deposited on a ultralene foil.

### Software

In order to develop, test and deploy different scanning strategies, major changes in the existing beamline end-station control software were required. Typical software for synchrotron XRM end-stations allows only for raster rectangular acquisitions with few exceptions on Ptychography setups, but even those have predefined sets of scan positions and acquisition parameters. A novel system was developed by engineers among the authors of this paper. It permits for non-regular positions and dynamic parameters that can all be changed during the scan on per-pixel basis. This system includes an advanced workflow manager (DonkiOrchestra^13^) which allows for easy and flexible changes of the data acquisition process. Other institutes are using similar systems (see Bluesky [https://nsls-ii.github.io/bluesky/ accessed: 25/02/2020]) thus the implementation of the proposed techniques also in other facilities should be quite easy. The data were stored in HDF5 files of non-standard structure and were later reconstructed in traditional dense data structures that can be stored in common formats (i.e. NeXus) and processed with popular analysis software (e.g. PyMca^17^). Both the acquisition framework and the reconstruction software were done in Python. The experimental data and their reconstructions are available in a public repository [10.5281/zenodo.3688316] respecting the FAIR principles.

## Supplementary information

Supplementary Information.
