# Supplementary material for: Compressive Sensing for Dynamic XRF Scanning
Source: Sci Rep. 2020 Jun 19;10:9990. doi: 10.1038/s41598-020-66435-6 (PMC7305138; doi:10.1038/s41598-020-66435-6)
Supplement: Supplementary file 1 — Supplementary Information. [file 41598_2020_66435_MOESM1_ESM.docx]

**Supplementary Information**

**Compressive Sensing for Dynamic XRF Scanning**

George Kourousias, Fulvio Billè, Roberto Borghes, Antonio Alborini, Simone Sala, Roberto Alberti and Alessandra Gianoncelli

**
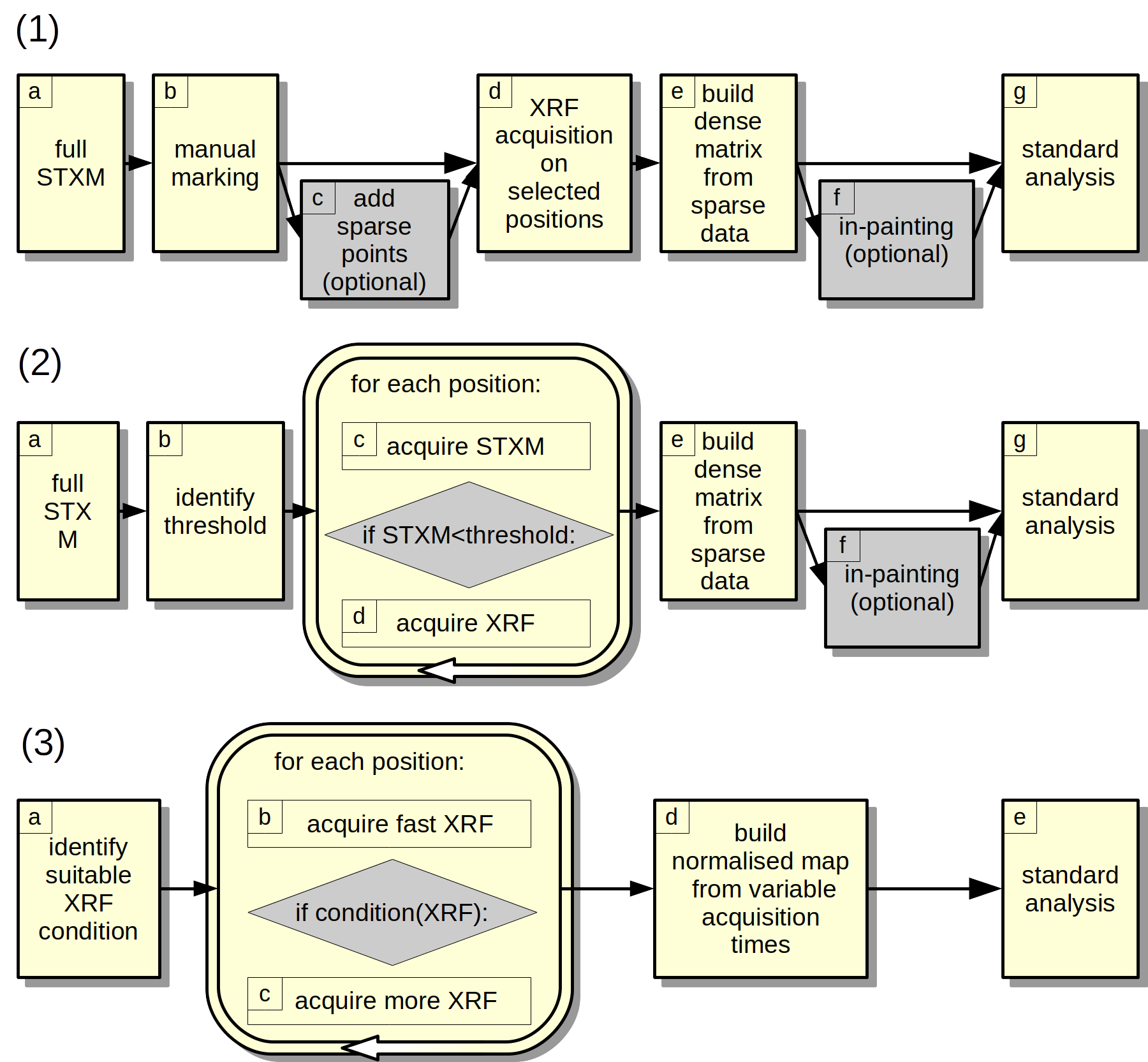
**

**S1.** *Schematic workflows for (1) Sparse scans and Masking, (2) Conditional and Multimodal acquisitions, and (3) Dynamic scans.*

A procedural description of the methods presented in this manuscript may provide an overview of the workflow and assist their implementation in other beamlines. The *sparse scans and masking* method (Results and Methods, section 1) requires a full STXM scan (s1.1a) which is used for the spatial selection of a suitable sub-ROI. This manual selection may be irregular and in TwinMic is done with a Wacom graphics tablet. Optionally, a suitable sparse pattern can be added outside the ROI in order to assist the in-painting/filling-the-missing-points method (s1.1c). The ROI, possibly expanded by the sparse pattern, results in a reduced set of scan points used by the acquisition system leading to a sparse set of XRF data (s1.1d). These data through computation are suitably reconstructed in a regular matrix (s1.1e) of the scanned area but including the missing values (Fig1e). Optionally, a suitable in-painting method (s1.1f) is used to reconstruct those missing values. The data at this stage can be further processed with any typical XRF workflow (s1.1g) thus calibrated, filtered, fitted, quantised etc.

The following workflow (s1.2) is for *conditional scans and multimodal acquisitions* (Results and Methods, section 2) where a secondary imaging technique is the decisive factor for XRF acquisition. Like for the previous workflow, it starts with an STXM acquisition (s1.2a) but its purpose is not to identify valid positions but only an x-ray transmission threshold (s1.2b). The threshold should discriminate the sample from its support or identify the most significant absorbing areas. The threshold can be derived from a representative area of the sample and then applied to a much larger scan. Once the threshold has been selected, for each position, the transmission signal is acquired (s1.2c) and according to a conditional comparison with the previously established threshold, a longer XRF acquisition is triggered or not (s1.2c). This results in a sparse set of data that are reconstructed to a matrix which may be in-painted and subsequently analysed with standard methods like in the previous workflow (s1.2e,f,g).

The final and most challenging workflow of dynamic scans (s1.3) (Results and Methods, section 3), does not require an additional technique like STXM (s1.1a, s1.2a) but assumes a suitable XRF condition (s1.3a) like a simple XRF signal threshold for a specific chemical element (as used in the presented results in Figure 3) or an intelligent ratio between different peaks etc. Following this, for each position in the scanned area, a fast XRF acquisition (s1.3b) is used together with the previously mentioned criteria (i.e. Na XRF levels) to decide dynamically whether to acquire a longer XRF signal (s1.3c) on the same position for better statistics. This results in a set of XRF data collected at variable exposure times that after a suitable normalisation can be reconstructed to a regular matrix (s1.3d).

Note that these three generic workflows can be easily combined resulting in an adaptable acquisition scheme.


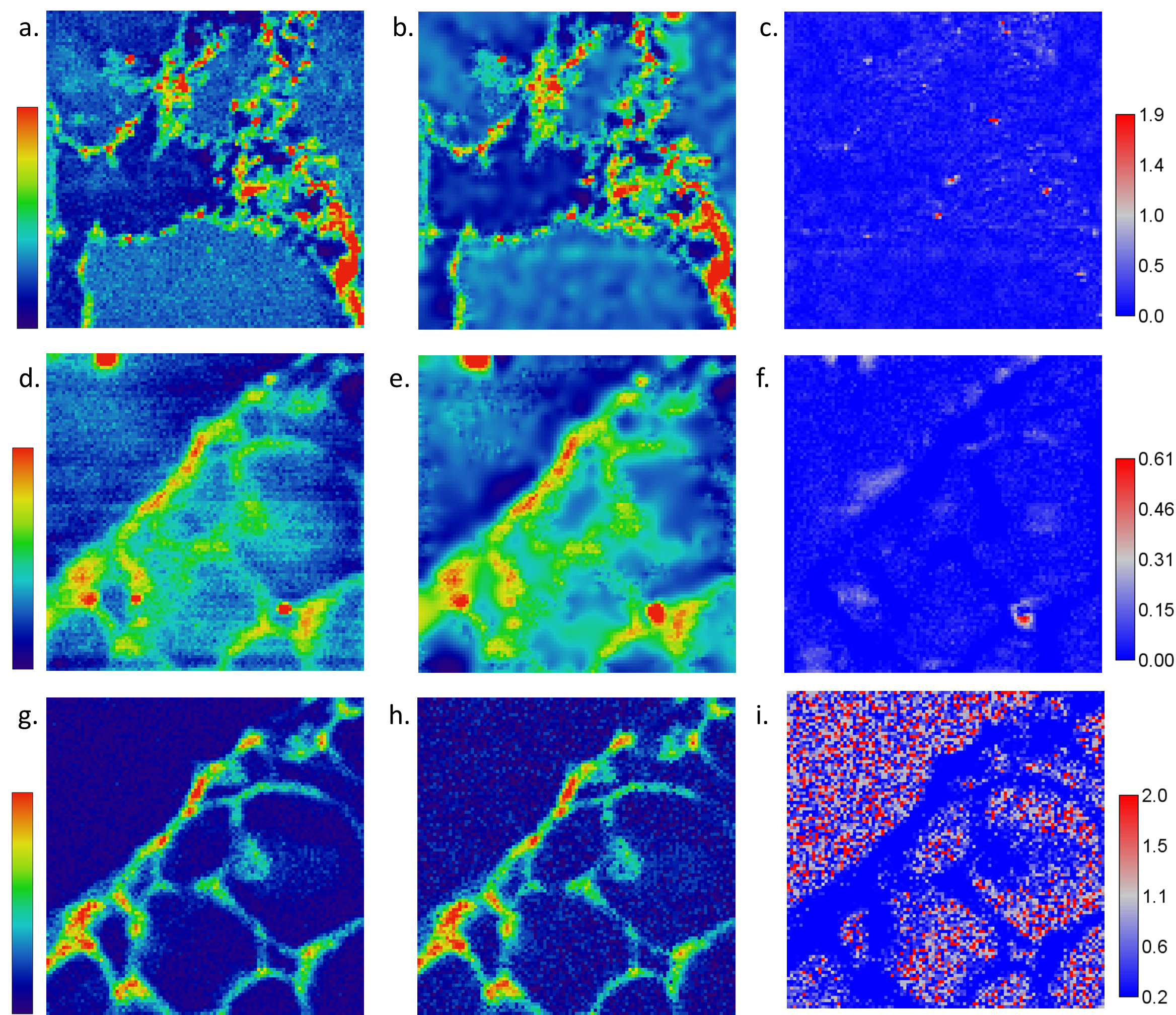


**S2.** *Reconstructed image evaluation of error. Relative error (c,f,i) between full XRF maps (a,d,g) and their corresponding reconstructions (b,e) from sparsely acquired data or dynamically varying acquisition (h). Panels (a,b,c) supplement Figure 1 on Sparse Scans and Masking, while (d,e,f) supplement Figure 2 on Conditional and Multimodal Acquisitions. Panels (g,h,i) refer to Figure 3 on Dynamic Scans where (g) is a full constant time acquisition and (h) is a variable one with better statistics in the regions of interest. The Error (c,f,i) is calculated as* |recostructed-original|/original*.*

The suggested methods as presented in their workflows consist of steps like those of mask definition, threshold identification and choice of signal logic (s1.1b,s1.2b,s1.3a) in combination with the reconstruction methods of XRF maps from sparse data (s1.1-2e-f, s1.3d-e). These steps can impact the quality of the reconstructed XRF map. On top of that, factors like mechanical instabilities (motor imprecisions and thermacal drifts) and x-ray beam fluctuations (position and intensity) add to the problem. Note that the final quality of this lossy process needs to be as high as possible aiming at reducing the losses at specific regions of interest while acquiring reduced data. Other than the encouraging empirical evaluation by an expert, a basic error measurement technique was proposed during the refereeing of this manuscript. The error is calculated as |reconstructed-original|/original. Other evaluation approaches have been implemented as well, including operating in logarithmic scales, pixel-wise standard deviation and structural similarity (SSIM), all of them resulting in comparable results. In specific, for data shown in Figure 1 representing the method of Sparse Scans and Masking the in-painted reconstruction (s2b) was compared to a typical full scan (s2a) resulting in an error map (s2c). Since these data, full and sparse (s2a,b) are collected subsequently and the mask is based on an initial full STXM acquisition (s1a), any sample stage instabilities create displacements that can be noticed after a pixel-wise comparison in the red values (>1) in their error map (s2c). Similarly, for the case of Conditional and Multimodal Acquisitions, for the maps of Figure 2 it was compared the in-painted reconstructed map (s2b) to the full one (s2a). While this technique is not requiring the *a priori* definition of a mask, it does require the identification of a transmission threshold (s1.2b). This makes it robust to mechanical instabilities since even if the sample is moved, the process decides point-by-point whether to acquire or not (s1.2c,d). This is also reflected on the error map (s2f) that is substantially lower than that of the Sparse scanning and Masking method (s2c). With a mean error of 0.026 and the qualitative evaluation of experience microscopy operator, it demonstrates a good case of compressive sensing.

An interesting remark from these experimental data is the error visible on the lower right section of the error map (in red) that results in a higher XRF signal on the reconstructed map (s2e) compared to the original one (s2d). After careful observation, it appears that this region has been mostly excluded from the STXM threshold (Fig2b,c,d) even though it has a strong XRF emission. This suggests that STXM may not always reveal the XRF regions of interest but also that the in-painting methods may overcompensate and introduce additional signal/artefacts through wrong interpolation.
